# Prader-Willi Syndrome: The Disease that Opened up Epigenomic-Based Preemptive Medicine

**DOI:** 10.3390/diseases4010015

**Published:** 2016-03-11

**Authors:** Takeo Kubota, Kunio Miyake, Natsuyo Hariya, Vuong Tran Nguyen Quoc, Kazuki Mochizuki

**Affiliations:** 1 Department of Epigenetic Medicine, Faculty of Medicine, University of Yamanashi, 1110 Shimokato, Chuo, Yamanashi 409-3898, Japan; kmiyake@yamanashi.ac.jp (K.M.); natsuyoh@yamanashi.ac.jp (N.H.); g15dhe04@yamanashi.ac.jp (V.T.N.Q.); 2 Department of Local Produce and Food Sciences, Faculty of Life and Environmental Sciences, University of Yamanashi, 4-4-37, Takeda, Kofu, Yamanashi 400-8510, Japan; mochizukik@yamanashi.ac.jp

**Keywords:** Prader-Willi syndrome, genomic imprinting, epigenetics, epigenomics, diagnosis, intervention, preemptive medicine

## Abstract

Prader-Willi syndrome (PWS) is a congenital neurodevelopmental disorder caused by loss of function of paternally expressed genes on chromosome 15 due to paternal deletion of 15q11–q13, maternal uniparental disomy for chromosome 15, or an imprinting mutation. We previously developed a DNA methylation-based PCR assay to identify each of these three genetic causes of PWS. The assay enables straightforward and rapid diagnosis during infancy and therefore allows early intervention such as nutritional management, physical therapy, or growth hormone treatment to prevent PWS patients from complications such as obesity and type 2 diabetes. It is known that various environmental factors induce epigenomic changes during the perinatal period, which increase the risk of adult diseases such as type 2 diabetes and intellectual disabilities. Therefore, a similar preemptive approach as used in PWS would also be applicable to acquired disorders and would make use of environmentally-introduced “epigenomic signatures” to aid development of early intervention strategies that take advantage of “epigenomic reversibility”.

## 1. Introduction

Prader-Willi syndrome (PWS) (OMIM#176270) is a congenital neurodevelopmental disorder characterized by neurocognitive deficits, excessive daytime sleepiness, muscle hypotonia, short stature, small hands and feet, hypergonadism, hyperphagia and obesity that leads to type 2 diabetes [1,2]. The genetic etiology of PWS is a 15q11–q13 deletion on the paternal chromosome 15, maternal uniparental disomy (UPD) of chromosome 15 or an imprinting mutation (micro-deletion encompassing the imprinting center) on the paternal chromosome 15, which cause a loss of function of paternally expressed genes on 15q11–q13 including *MKRN3*, *MAGEL2*, *NDN*, *SNURF-SNRPN*, and *SNORD116* snoRNA (HBII-85 C/D box small nucleolar RNA cluster) [3,4]. Recent studies demonstrated that small deletions of the paternal allele of the *SNORD116* snoRNA and mutations on the paternal allele of *MEGEL2* can cause PWS [5,6], suggesting that the phenotypic overlap between *SNORD116* deletion cases and *MAGEL2* mutation cases might be caused by changes in higher-order chromatin structure at the 15q11–q13 locus [6]. Furthermore, a recent study using induced pluripotent stem cells derived from the PWS patients revealed up-regulation of maternally expressed genes in the imprinted DLK1-DIO3 locus on chromosome 14 due to deficiency of the paternal allele of IPW, a long noncoding RNA at the 15q11–q13 locus that acts as a regulator of the DLK1-DIO3 region, and this indicates that a subset of PWS phenotypes may arise from dysregulation of an imprinted locus distinct from the PWS critical region of 15q11–q13 [7].

**Figure 1:**
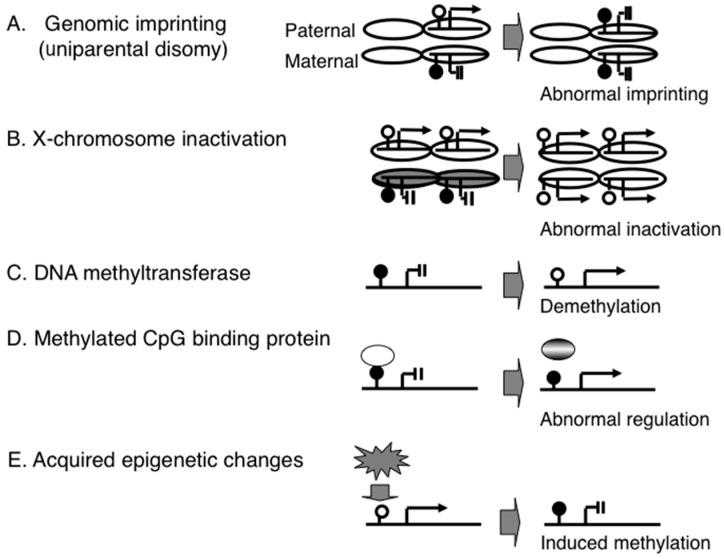
Epigenetic mechanisms associated with congenital and acquired disorders. (**A**) genomic imprinting and a congenital disorder (e.g., Prader-Willi syndrome); (**B**) X-chromosome inactivation and a congenital disorder (e.g., ring X Turner syndrome); (**C**) DNA methyltransferase and a congenital disorder (e.g., ICF syndrome); (**D**) methyl-CpG-binding protein and a congenital disorder (e.g., Rett syndrome); (**E**) epigenetic gene regulation system and an acquired disorder (e.g., type 2 diabetes).

PWS was one of the first human diseases that was caused by abnormal genomic imprinting. Genomic imprinting refers to a parental-of-origin specific gene expression based on epigenetic gene regulation that does not depend on DNA sequence but on DNA and histone chemical modifications. For example, the maternal allele of the promoter region of the *small nuclear ribonucleoprotein polypeptide N* (*SNRPN*) gene is hypermethylated and suppressed; by contrast, the paternal allele of this region is not methylated and expressed (Figure 1A) [8,9]. To date, a number of imprinted genes have been identified; the imprinting patterns on these genes have been shown to be variable among tissues, such as the brain and peripheral blood [10].

Another example that was caused by abnormal genomic imprinting in 15q11–q13 is Angelman syndrome (AS). In this syndrome, the direction of the imprinting is opposite to that of PWS: it is caused by is either a 15q11–q13 deletion on the maternal allele, paternal UPD15 or an imprinting mutation on the maternal chromosome 15. AS is characterized by severe intellectual disability, intractable epilepsy, puppet-like ataxic movement, and paroxysms of laughter. In contrast to PWS in which several genes are responsible for the phenotype, loss of function of *ubiquitin protein ligase E3A* (*UBE3A*) is involved in the pathogenesis of AS because a subset of AS patients showed point mutations of this *UBE3A* [11]. Interestingly, an increased copy number (*i.e.*, duplication or triplication) of the maternal 15q11–q13 region with increased *UBE3A* expression causes autistic-spectrum disorders [12], suggesting that both under- and over-expression of the gene can affect the brain function.

Genomic imprinting on a different chromosome (11p15.5) is involved in Beckwith–Wiedeman syndrome (BWS), an over-growth disorder characterized by macrosomia, macroglossia, visceromegaly, embryonal tumor. An increased copy number of the paternally expressed *insulin-like growth factor 2* (*IGF2*) gene due to uniparental disomy or duplication is associated with the over-growth phenotype [13]. Interestingly, aberrant DNA methylation of the 11p15.5 imprinted region leads to clinical syndromes with opposite growth phenotypes: BWS and Russell–Silver syndrome (RSS) with severe fetal and postnatal growth retardation [14]. Loss of function due to nonsense mutation in the paternal allele of *IGF2* also leads to intrauterine and postnatal growth restriction with RSS-like dysmorphic features [15].

A different epigenetic phenomenon is X-chromosome inactivation (XCI). This is not a single gene-based mechanism as in genomic imprinting but is a chromosome-level epigenetic mechanism. The mammalian X chromosome is much larger than the Y chromosome and carries many more genes. Thus, females (karyotype XX) have more genes than males (XY). This gene imbalance is compensated by the epigenetic inactivation of one of the two X chromosomes in females [16]. Since failure of XCI (functional X disomy) leads to abortion, as shown by mice produced by somatic cloning [17], the establishment of a proper XCI pattern in early development is essential to normal birth. However, if one of the two X chromosomes is extremely small (e.g., a small ring X chromosome), failure of XCI does not always lead to abortion; however, such live-born cases show severe congenital neurodevelopmental disorders (e.g., ring X Turner syndrome) [16]. This indicates that XCI is an essential epigenetic mechanism for normal brain development (Figure 1B).

To date, a number of proteins and enzymes involved in epigenetic gene regulation have been identified, and some of these have been shown to be associated with congenital neurodevelopmental disorders. For example, homozygous mutations of the *DNMT3* gene, which encodes a DNA methyltransferase, cause ICF syndrome, which is characterized by immunodeficiency, centromere instability and facial anomalies. The heterochromatic regions of chromosomes 1, 9 and 16 in ICF patients are demethylated and are prone to involvement in chromosome breaks and rearrangements [18]. Mutations of *DNMT3B* are thought to cause demethylation in gene promoter regions, leading to de-suppression of immunodeficiency-associated genes (Figure 1C). A recent study revealed that mutations in another DNA methyltransferase gene, *DNMT3A*, were found in the patients with an overgrowth syndrome and intellectual disability [19].

Another well-studied epigenetic disorder is Rett syndrome, which is characterized by epileptic seizures, ataxic gait and autistic behavior. This syndrome is caused by mutation of the X-linked *MECP2* gene: patients are all female and are heterozygous for the mutation, since the mutation is an embryonic lethal condition in males. *MECP2* encodes a protein that has a methylated-CpG binding domain, and mutation leads to de-suppression of genes associated with neurological features (Figure 1D) [20,21].

As mentioned above, following the initial characterization of the epigenetic basis of PWS, studies on other human diseases have identified further examples of epigenetic phenomena. It is now known that epigenetic effects have a role in congenital neurodevelopmental disorders and adult-onset acquired diseases. This understanding of the scope of epigenetic effects will be valuable for developing medical treatment strategies in the future. In this article, we introduce epigenomic-based diagnostic assays developed from the accumulated knowledge of acquired epigenomic changes associated with various diseases. These assays were initially developed in studies on PWS and offer the possibility of preemptive medicine (early detection and early intervention) based on epigenomic diagnostic markers.

## 2. Development of Diagnostic Assays for PWS

### 2.1. Diagnostic Assays for PWS

**Figure 2:**
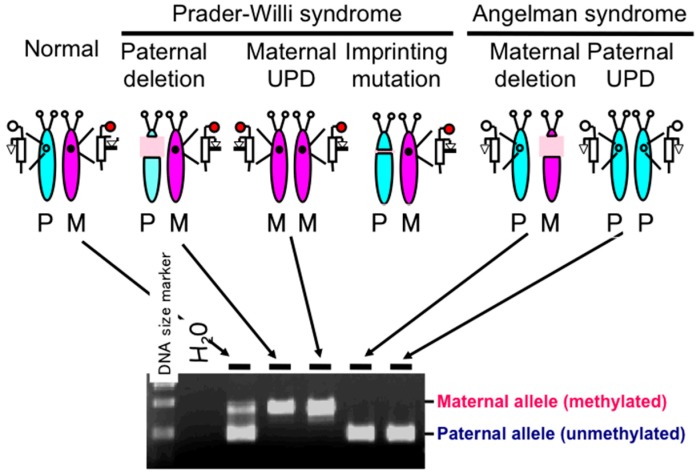
Genetic etiology of Prader–Willi syndrome (PWS) and results of methylation-specific PCR assays of the promoter region of the small nuclear ribonucleoprotein polypeptide N (*SNRPN*) gene [9]. The assay can detect all three etiologies of PWS, namely, paternal deletion, maternal uniparental disomy (UPD) and imprinting mutation (microdeletion encompassing the imprinting center). Results from an imprinting mutation case are not illustrated here. The assay also detects Angelman syndrome with maternal deletion and paternal UPD. P: paternal chromosome 15, M: maternal chromosome 15.

PWS patients were initially classified into three genetic categories, namely, paternal deletion, maternal UPD, and imprinting mutation (Figure 2). Detection of paternal deletion was originally performed using high resolution chromosome banding; however, this method has now been replaced with a more sensitive method, fluorescence *in situ* hybridization (FISH) [22]. Maternal UPD is identified using dinucleotide (CA repeat) polymorphic markers that distinguish between the paternally and maternally derived chromosome 15 [23].

### 2.2. DNA Methylation-Based Diagnostic Assay for PWS

Beside the assays described above, a DNA methylation-based diagnostic assay was developed, which can detect the rare cases of an imprinting mutation. This protocol combines Southern blot assays with use of methylation-sensitive restriction enzymes (e.g., *Hpa*II) to detect aberrant hypermethylation in multiple loci within 15q11–q13 due to the malfunction of the imprinting center. Since a deletion or UPD can also produce an aberrant hypermethylation pattern in the 15q11–q13 loci, the DNA methylation assay is capable of identifying these causes of PWS as well as those with an imprinting mutation. Thus, the assay became a standard diagnostic assay for PWS as it could detect all three major categories [8]. However, the Southern blot-based assay requires one week to obtain results and make use of radioisotopes; thus, it was not a suitable method for diagnostic laboratories in hospitals. Instead, a rapid and reliable PCR-based assay was desired. We developed such an assay based on a methylation-specific PCR technique initially used to identify aberrant methylation patterns in cancer [24]. This PCR-based PWS methylation assay is widely used in diagnostic laboratories [9] (Figure 2). Recently, methylation-specific multiplex ligation-dependent probe amplification (MS-MLPA) assay is also used for diagnosis of PWS, which can detect not only all three major categories described above but also microdeletions at the imprinting center and *SNORD116* regions and rare duplications [25,26,27].

### 2.3. DNA Methylation-Based Diagnostic Assay for other Congenital Neurodevelopmental Disorders

The PCR-based methylation assays were developed for diagnosis of other congenital neurodevelopmental disorders with aberrant methylation patterns, such as Fragile X and UPD14 syndromes [28,29,30].

### 2.4. Genome-Wide Epigenetic Assay

In recent years, the technology for DNA-based microarrays has advanced rapidly; this methodology can identify genome-wide genetic changes, such as altered gene expression patterns, single-nucleotide polymorphisms, and copy-number variations. Microarray analysis also allows identification of genome-wide epigenetic changes (epigenomic changes). Using this approach, we assessed the extent of X-chromosome inactivation in a derived chromosome 15 in a patient with a t(X;15) translocation and a partial PWS phenotype [31]. We also used this method to identify differential DNA methylation between monozygotic twins in order to determine the basis of discordant neurological features [32]. Environmentally-induced epigenomic changes, such as tobacco smoke-induced reduction in DNA methylation, have also been identified using beadchip technology and genome-wide DNA methylation screening [33].

## 3. Acquired Epigenomic Changes Associated with Diseases

### 3.1. Epigenomic Changes in Cancer

As described above, the PCR-based methylation assay was initially developed for cancer studies [24], as epigenetic changes have been identified in many cancer samples. Therefore, cancer epigenomics has become one of the major research fields in medical epigenomics. However, it is not clear whether epigenetic changes are the initial events (causes) of carcinogenesis, as genetic changes are simultaneously observed in cancer tissues; mutations (genetic changes) of gene encoding proteins such as DNA methyltransferases and MBD proteins might secondarily induce changes in DNA methylation or histone modifications (epigenetic changes) in cancer cells. In this context, a recent study demonstrated a case of ependymoma (one type of childhood brain tumors) caused by epigenetic changes without any mutations [34], suggesting that epigenetic changes can be the initial event in carcinogenesis and indicating that abnormal establishment of the epigenomic pattern may underlie carcinogenesis during early development.

### 3.2. Environmental Factors that Induce Epigenomic Changes

It was initially believed that epigenetic changes were observed only in cancers and congenital disorders such as PWS. However, recent evidence suggests that epigenetic changes may also be involved in environmentally-induced acquired diseases: various environmental factors are now known to alter epigenetic patterns and subsequently to change gene expression. These include folic acid (an essential nutrient for methylation) [35], royal jelly [36], drugs for psychiatric diseases [37,38,39], environmental chemicals [40,41], tobacco smoking [42], external stimuli (electro-convulsive treatment for psychiatric diseases) [43], and assisted reproductive technologies (e.g., *in vitro* fertilization and intracytoplasmic sperm injection) [44,45]. The latter has been suggested to be associated with an increased risk of genomic imprinting disorders including PWS, although this supposed connection is controversial [46,47].

### 3.3. Environmental Stresses May Induce Epigenetic Changes during the Neonatal Period

In addition to the various environmental factors listed above, it has been suggested that mental stress in early life can also alter the epigenetic state in the brain. Neonatal rats that were subjected to short-term mental stress by reducing maternal care in the first week of life showed increased DNA methylation in the promoter region of the glucocorticoid receptor (*GR*) gene (also known as the nuclear receptor subfamily 3 group C member 1, *NR3C1*) in the hippocampal region. This increased methylation caused prolonged low expression of the gene that is associated with persistent abnormal behavior. By contrast, promoter methylation was decreased in the hippocampal region of offspring of high maternal care mothers [48]. This phenomenon has been suggested to provide an animal model of the long-term effect of childhood neglect and maltreatment in humans. There is some evidence in humans that is consistent with this finding in rats; postmortem analysis of the hippocampus of suicide victims with a history of childhood abuse identified hypermethylation of the neuron-specific promoter region of *GR* in combination with a decrease in gene expression [49]. This finding suggests that the adverse effects of early-life stress may last throughout life with aberrant DNA methylation in the brain [50] and also indicates that neurodevelopmental problems not only arise from congenital genetic or epigenetic abnormalities but also from acquired epigenetic dysregulation caused by environmental factors in early life.

### 3.4. Environmental Stresses Induce Epigenetic Changes during Fetal Development

Poor nutrition can cause changes in the epigenomic state during fetal development. For example, a calorie or protein-restricted maternal diet can decrease DNA methylation of energy-storage genes, such as peroxisome proliferator-activated receptor alpha (*PPARα*) in the fetal liver; this altered methylation status induces *PPARα* expression and results in a “thrifty phenotype” that is suitable for survival under poor nutritional conditions after birth [51,52]. Recent human epidemiological studies suggest that the “thrifty phenotype” induced by either poor nutrition during fetal development or the self-imposed maternal dieting during pregnancy that is popular among young women in industrial countries, increases the postnatal risk of non-communicable diseases (NCDs), which are chronic, non-infectious conditions of long duration (e.g., obesity, type 2 diabetes, intellectual disabilities and neurodevelopmental disorders) [53,54,55]. The increased risk of NCDs is the result of a mismatch between the programmed nature of the fetus to survive adverse conditions after birth and the actuality of life in an affluent society. This is a central concept of the recently established research field of “Developmental Origins of Heath and Disease (DOHaD)” [56].

Recent studies suggest that over-nutrition also change the epigenome of the fetus and the gamete. For example, high-fat diet-fed obese males have higher DNA methylation at satellite repeats throughout the genome in rats [57], newborns of obese fathers have lower DNA methylation at multiple imprinted genes in the umbilical cord blood leukocytes in humans [58,59], spermatozoa from obese men carry a distinct DNA methylation pattern compared to lean men, in particular at protamine-bound regions compared to histone-retained regions, which includes genes related to appetite control (*MC4R*, *BDNF*, *NPY*, *CR1*, and *CART*) and metabolism (*FTO*, *CHST8*, *SH2B1*) [60], and spermatozoa from obese males carry distinct mRNA and microRNA profiles that are potentially associated with partial transmission of metabolic disturbances such as obesity and type 2 diabetes in two generations in mice [61]. These results suggest that paternal lifestyle or over-nutrition influences the reprogramming of epigenetic marks during spermatogenesis.

## 4. Epigenomic-Based Preemptive Medicine

### 4.1. Therapeutic Strategies and Implications of Early Intervention for PWS

For PWS patients, special feeding techniques, such as gavage feeding for the first several weeks of life, is effective to assure adequate nutrition and avoid failure to thrive. A program of a well-balanced low-calorie diet, regular exercise and close supervision to minimize food theft is effective for the hyperphagia and weight gain that begins at 2–4 years of age to prevent obesity and its consequences [62]. Physical therapy for under three-year-olds years improves muscle strength and encourages achievement of developmental milestones, and daily muscle training increases physical activity and lean body mass in older patients [63]. Growth hormone (GH) treatment normalizes height, increases lean body mass, decreases fat mass, and increases mobility, which are beneficial to weight management [64]; a longitudinal study demonstrated that GH treatment normalized stature and improved weight and body composition in PWS compared with non-growth hormone-treated PWS individuals [65,66]. Furthermore, a recent randomized controlled trial revealed that physical training combined with GH increased muscle thickness, which was matched by an increase in muscle strength and motor development in infants with PWS [67].

GH treatment can be started in infancy or at the time of diagnosis [68], and it improves language skills in infancy, cognitive skills in childhood, and mental speed, mental flexibility, motor performance and ability to adapt to society in adulthood [69,70,71,72]. Therefore, early intervention based on early diagnosis is strongly recommended for individuals with PWS.

### 4.2. Epigenomic-Based Preemptive Medicine

In NCDs or adult diseases, doctors make a diagnosis based on the guidelines established for each disease; however, diagnosis is often made at a rather late stage of development or because diagnosis is postponed until the patient fulfills the criteria of each adult disease (e.g., blood sugar and HbA1c levels for type 2 diabetes). Furthermore, gold standard therapeutic protocols are strictly determined in the guidelines, regardless of the patient’s individual genetic background that may influence the effectiveness of drugs and usefulness of surgery. The basis for “personalized medicine” is the application of treatments that take into consideration each patient’s genetic background.

“Preventive medicine” is population-based and started as a means for preventing infectious diseases; it is now moving toward prevention of NCDs. Preventive medicine will revolutionize healthcare systems by altering the emphasis from reactive therapies to preventive treatments—in other words, from waiting until the patient is sick to assessing healthcare needs prior to disease onset [73].

**Figure 3:**
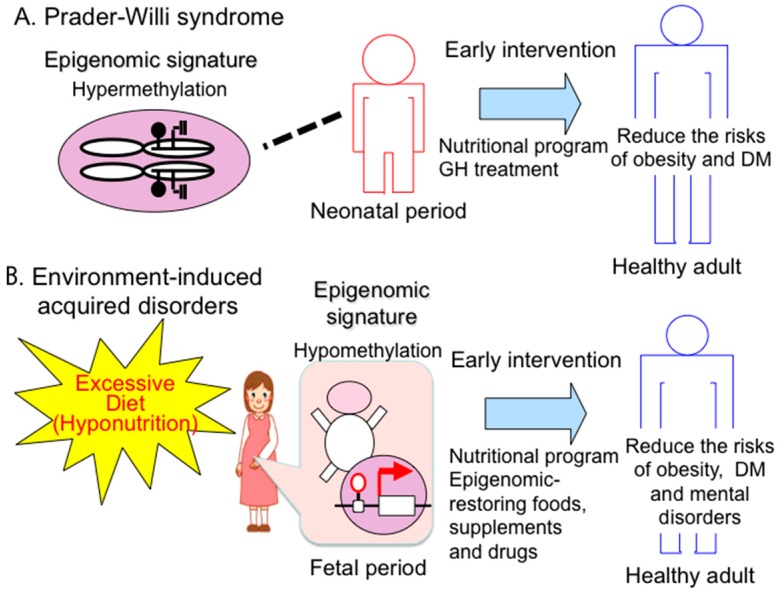
Preemptive medicine in congenital and acquired disorders. (**A**) Prader-Willi syndrome. Early diagnosis based on a specific epigenomic (DNA methylation) pattern has recently enabled early intervention such as an education nutritional program and GH therapy to prevent from expected complications (e.g., obesity, type 2 diabetes mellitus (DM)); (**B**) acquired disorders—early detection of epigenomic changes induced by hyponutrition stress will enable early intervention, such as an education nutritional program and epigenome restarting drugs to prevent expected complications (e.g., obesity, DM).

“Preemptive medicine” is a type of “personalized medicine” that is based on the individual and thus is different from population-based preventive medicine. In preemptive medicine, a practical approach is to detect high-risk individuals by (epigenome-based) blood biomarker screening, and to follow up this screening with early intervention at the preclinical stage to prevent serious events, such as type 2 diabetes, Alzheimer’s disease, osteoporosis and coronary heart disease. A preemptive approach has already been established for PWS. High-risk individuals (*i.e.*, PWS patients) are identified by epigenomic-based blood testing in infancy, and a variety of physical and drug treatments are provided to the individuals to prevent future symptoms (e.g., obesity, type 2 diabetes) (Figure 3A).

In order to establish an intervention program for acquired disorders similar to that for PWS, it will be necessary to identify “epigenomic signatures” that are induced by environmental factors and can be detected in peripheral blood. In fact, mental stress-induced hypermethylation of the *brain-derived neurotrophic factor* (*BDNF*) gene was demonstrated in the hippocampal region in a mouse model of depression [37], and abnormal DNA methylation of *BDNF* in peripheral blood is subsequently shown in the individuals with major depression [74]. Furthermore, bisphenol A (BPA), an environmental chemical, exposure during prenatal period induced lasting DNA methylation changes in *Bdnf* in the mouse hippocampus and blood, and high BPA exposure *in utero* induced DNA methylation changes in the human cord blood [75]. These findings suggest that *BDNF* DNA methylation in the blood may be used as a predictor of brain DNA methylation and further indicate that DNA methylation in the peripheral blood can be a useful biomarker for the detection of psychopathology.

Taking advantage of the use of epigenomic reversibility, it is feasible to develop effective treatments with appropriate nutrients and chemicals (e.g., folic acid or histone deacetylase (HDAC) inhibitors) to restore the impaired epigenomic changes induced by environmental factors [76,77,78,79,80]. In this epigenomic context, drugs are under development by many pharmacological companies for such therapeutic purposes.

## 5. Conclusions

PWS is a unique and important disorder that has opened up a novel epigenetic paradigm and a novel early intervention. PWS was one of the first disorders to be identified as caused by epigenetic abnormalities and is a representative disease in which early intervention improves the quality of life of affected individuals. Such medical approaches can be achieved through use of an effective diagnostic assay to detect the specific epigenomic patterns of all types of PWS.

Epigenomic patterns can be altered by various environmental factors, especially in the perinatal period. In this case, changes in epigenomic patterns are modest and variable in contrast to the all-or-none methylation difference between the maternal and paternal alleles in the imprinted loci**.** Altered epigenomic patterns can lead to abnormally persistent gene expression and can increase disease risk through adverse influence on health in later life. Therefore, an effective diagnostic assay to detect specific epigenomic alterations that predict acquired diseases, such as type 2 diabetes and intellectual disabilities, needs to be developed to identify high-risk groups. The early prediction of latent diseases allows early intervention, and such preemptive treatments are the ultimate goal of medicine (Figure 3B).
